# Artificial intelligence in nephrology education: a multicenter survey of fellowship trainees at Mayo Clinic

**DOI:** 10.3389/fneph.2025.1607017

**Published:** 2025-06-18

**Authors:** Mohammad S. Sheikh, Charat Thongprayoon, Iasmina M. Craici, Jing Miao, Fawad M. Qureshi, Michael A. Mao, Musab S. Hommos, Mary Prendergast, Sumi Nair, Kianoush B. Kashani, Wisit Cheungpasitporn

**Affiliations:** ^1^ Division of Nephrology and Hypertension, Mayo Clinic Minnesota, Rochester, MN, United States; ^2^ Division of Nephrology and Hypertension, Mayo Clinic Florida, Jacksonville, FL, United States; ^3^ Division of Nephrology and Hypertension, Mayo Clinic Arizona, Phoenix, AZ, United States

**Keywords:** artificial intelligence, nephrology fellowship, educational needs, clinical decision-making, fellowships, health knowledge

## Abstract

**Background:**

Artificial intelligence (AI) is increasingly recognized for its potential to enhance nephrology training and practice. However, the integration of AI into fellowship training remains inadequately explored. This study aimed to assess current AI utilization, perceptions, and educational needs among nephrology fellows at Mayo Clinic.

**Methods:**

A structured online survey was administered to 23 fellows—including those specializing in kidney transplantation and onco-nephrology—across three Mayo Clinic sites (Minnesota, Arizona, and Florida). The survey addressed domains such as current AI usage, perceived relevance of AI in clinical practice, interest in formal AI training, self-assessed comfort with AI integration, and barriers to adopting AI technologies in nephrology education.

**Results:**

A total of 21 fellows (91% response rate) participated in the survey. 76% of respondents rated AI as moderately to highly relevant to nephrology. Similarly, 76% indicated a moderate to very high interest in receiving targeted AI training. Despite these favorable perceptions, 76% had rarely or never used AI in their clinical or research activities, and none reported any formal AI education. Interactive workshops emerged as the preferred modality for AI training (52%), with limited knowledge cited as the primary barrier to adoption. Optimism was especially high regarding AI applications in predictive modeling (86%) and diagnostic imaging (81%), while confidence in AI for direct clinical decision-making remained cautious.

**Conclusion:**

There is significant interest among nephrology fellows in AI, along with a critical need for formal education and training. The enthusiasm for AI’s potential contrasts with a cautious perspective towards its current use in clinical decision-making. Our study highlights the necessity for educational initiatives that bridge the knowledge gap and foster confidence in the appropriate use of AI technologies in Nephrology fellowship.

## Introduction

Artificial Intelligence (AI) is rapidly transforming the practice of medicine, promising enhanced precision, efficiency, and predictive capabilities (1, 2). Nephrology, the field addressing kidney health and disease, is uniquely positioned to benefit from AI-driven innovations (3). However, the integration of AI into nephrology practice depends not only on technological breakthroughs but also on the readiness and adaptability of emerging nephrologists (4–6). Understanding nephrology fellows’ perspectives on AI is key to designing effective educational programs (6).

Recent research has focused on leveraging machine learning algorithms to predict the progression of chronic kidney disease (CKD), detect acute kidney injury, and optimize dialysis treatments (7–9). When these AI tools are integrated with actionable preventive strategies, they have the potential to significantly improve patient outcomes, lower healthcare costs, and streamline nephrology practices (10, 11). Nonetheless, a substantial gap remains between AI’s theoretical promise and its practical application, largely due to limited exposure and training among current nephrology fellows (12).

Assessing the interest and attitudes of nephrology fellows towards AI is essential for tailoring educational programs that align with their learning needs and professional aspirations. By evaluating their enthusiasm, identifying perceived barriers, and determining preferred learning modalities, fellowship programs can develop AI-focused curricula to enhance nephrology training (13). These initiatives may include integrating AI modules into existing curricula, offering immersive hands-on workshops, and fostering collaborations with AI experts. Furthermore, analyzing the factors that shape fellows’ interest in AI can illuminate deficiencies in the current educational framework. For instance, if fellows express concerns regarding insufficient AI-related resources or mentorship opportunities, fellowship programs can proactively address these shortcomings by enhancing access to up-to-date, expert-driven resources. By nurturing a culture of innovation and equipping fellows with the requisite tools and guidance, fellowship programs can cultivate a new generation of AI-literate nephrologists who are poised to harness AI’s potential safely and effectively.

This study evaluates the current state of AI utilization among nephrology fellows at Mayo Clinic campuses in Minnesota, Arizona, and Florida by examining their experiences, identifying barriers to adoption, and exploring strategies to address educational gaps that facilitate the successful integration of AI into their future clinical practice. By comprehensively understanding fellows’ perspectives, we can identify and overcome potential roadblocks, and subsequently design tailored educational programs that address their unique needs. Moreover, the findings of this study may have broader implications beyond education. Gauging the interest of nephrology fellows in AI provides insights into its potential transformative impact on the entire field of nephrology. High levels of enthusiasm for AI among fellows may signal a broader shift toward AI-driven innovation in nephrology, potentially attracting new talent and expediting the development of advanced diagnostic and therapeutic tools (14). Conversely, significant reservations or concerns regarding AI may underscore the necessity for intensified outreach and educational efforts to better elucidate its benefits in nephrology.

## Methods

### Study design and participants

This study utilized a cross-sectional survey design to assess the current state of AI utilization, perceptions, and educational needs among nephrology fellows at the Mayo Clinic. The survey targeted nephrology fellows, kidney transplant fellows, and onconephrology fellows from the Mayo Clinic’s three main campuses located in Minnesota, Arizona, and Florida. The study was approved by the Institutional Review Board (IRB) of the Mayo Clinic (ID: 23-012245), ensuring compliance with ethical standards for research involving human subjects.

### Survey development

The survey instrument was developed collaboratively by a team of nephrologists, AI specialists, and medical educators to ensure content validity and relevance (**Supplementary Material**). The survey comprised four main sections. This consisted of 1) demographic information to collect basic information such as age, year of fellowship, and subspecialty track, 2) current use of AI to evaluate the frequency and context of AI use in clinical practice and research activities, with respondents asked to specify the frequency they have used AI-based tools and to describe the purposes for which these tools were employed; 3) perceptions of AI in nephrology to explore fellows’ opinions on the relevance of AI to their field, their confidence in using AI for clinical decision-making, and their views on the potential benefits and risks associated with AI in nephrology; and 4) educational needs and barriers to focus on fellows’ interest in AI training, preferred formats of learning (e.g., workshops, seminars, online modules), and perceived obstacles to integrating AI into their professional practice.

The survey questions utilized a mix of multiple-choice, Likert scale, and open-ended responses to capture a broad spectrum of responses.

### Survey distribution and collection

The survey was distributed using REDCap (Research Electronic Data Capture), a secure, web-based platform hosted at Mayo Clinic that supports data capture for research studies. A master list of eligible participants was generated by the fellowship coordinators from each campus. An initial email invitation containing a personalized REDCap survey link and an IRB-approved information sheet was sent by a neutral administrative coordinator unaffiliated with the research team to ensure impartiality. This initial message emphasized that participation was voluntary, that responses would be anonymous, and that no identifying information (such as IP address or email metadata) would be collected. The REDCap system was configured to prevent collection of any respondent-identifiable data, and each participant could only submit one response. Two reminder emails were sent at 10-day intervals by the same coordinator. The data were stored in a password-protected REDCap database accessible only to the principal investigator and designated statistical analyst, both of whom were trained in human subjects research protection. No incentive was offered for participation.

### Data Analysis

After survey closure, data were exported from REDCap into JMP Pro 17 statistical software (SAS Institute, Cary, NC). Descriptive statistics were used to characterize demographic data and survey responses. Categorical data (e.g., Likert scale responses) were summarized as frequencies and percentages. No imputation was performed for missing responses; only completed surveys were included in the final analysis. Subgroup analyses (e.g., by fellowship year or location) were not performed due to the limited sample size, but may be explored in future expanded studies. The analysis approach was determined *a priori* and aligned with accepted practices for survey-based educational needs assessments.

## Results

### Response rate and demographics

Of the 23 nephrology fellows solicited to participate across Mayo Clinic’s campuses in Minnesota, Arizona, and Florida, 21 (91%) completed the survey. **Table 1** shows the characteristics of respondents in this survey.

**Table 1:** Characteristics of Nephrology Fellows Participating in the Survey (n = 21).

Characteristic	n (%)
Age Group	
25–28 years	2 (10%)
29–32 years	6 (29%)
33–36 years	9 (43%)
37–40 years	1 (5%)
>40 years	3 (14%)
Fellowship Year	
First-year	8 (38%)
Second-year	4 (19%)
Third-year	4 (19%)
Onconephrology Fellow	1 (5%)
Transplant Fellow	4 (19%)
Fellowship Location	
Minnesota	13 (62%)
Arizona	3 (14%)
Florida	5 (24%)

Age in years. Values are presented as number (percentage).

### Perceptions of AI relevance

**Figure 1:**
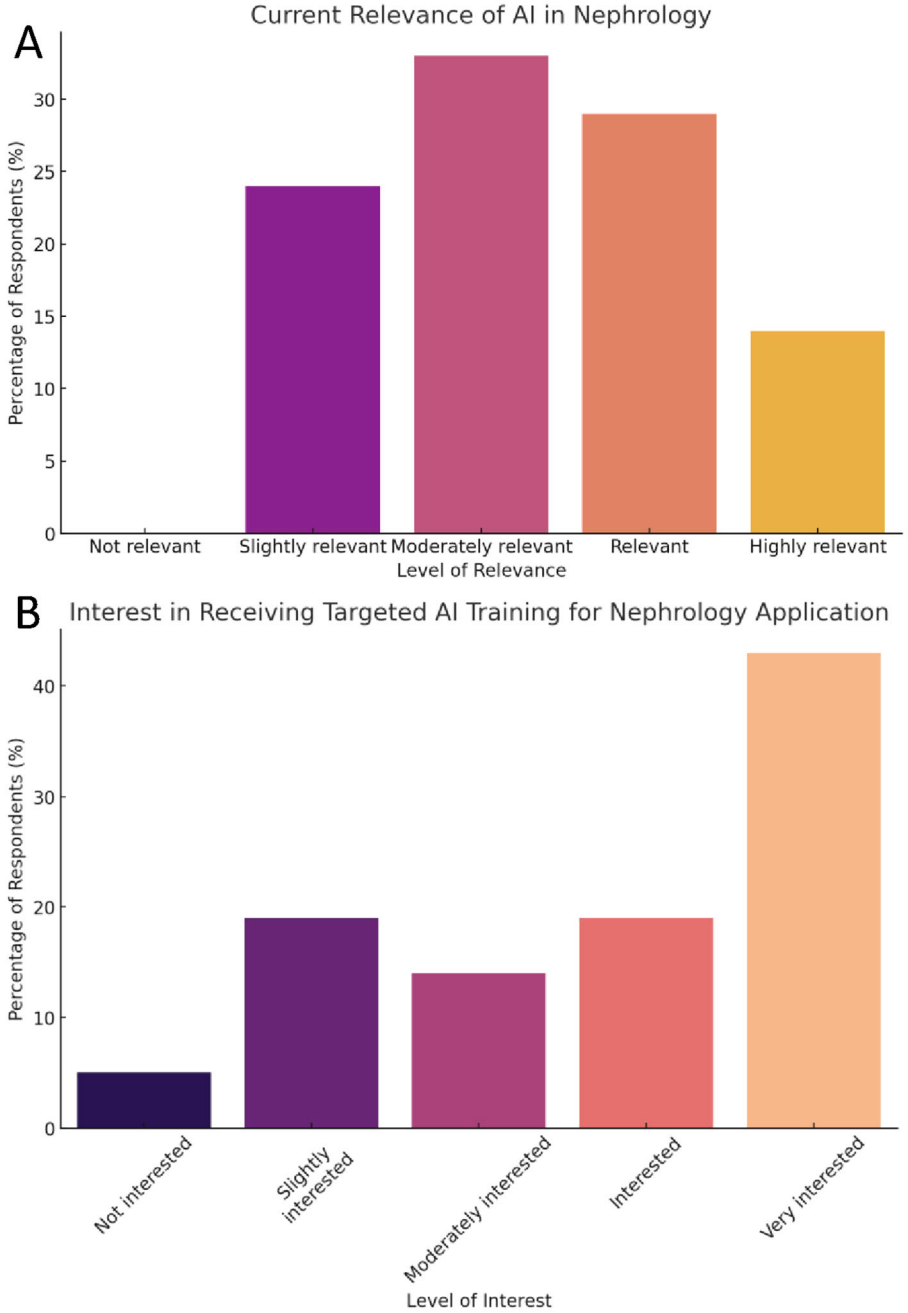
Fellows’ perceptions of AI in nephrology and interest in training. **(A)** Bar chart showing the percentage of respondents (n=21) who rated AI’s relevance in nephrology on a five-point Likert scale ranging from “Not relevant” to “Highly relevant.” **(B)** Bar chart displaying levels of interest in receiving targeted AI training, categorized from “Not interested” to “Very interested.” Percentages represent total respondents selecting each option.

The majority (76%) of the respondents acknowledged the relevance of AI in their field, rating it as moderately to highly relevant (**Table 2**, Question 1). This indicates a strong recognition within the nephrology community of the potential applications and benefits of AI technologies in enhancing clinical practice and research (**Figure 1A**). Most respondents felt comfortable with the idea of integrating AI to assist with administrative tasks (62%) and knowledge acquisition (67%). Still, a smaller proportion (43%) felt comfortable with the idea of integrating AI into clinical practice (**Table 2**, Question 2).

**Table 2:** Survey responses on AI use, perceptions, and educational needs (n = 21).

Survey question	Response options	n (%)
1. Relevance of AI in Nephrology	Not relevant	0 (0%)
	Slightly relevant	5 (24%)
	Moderately relevant	7 (33%)
	Relevant	6 (29%)
	Highly relevant	3 (14%)
2. Comfort witd Integrating AI into Daily Practice		
*Clinical Practice*	Uncomfortable	2 (10%)
	Neutral	10 (48%)
	Comfortable	9 (43%)
*Knowledge Acquisition*	Uncomfortable	2 (10%)
	Neutral	5 (24%)
	Comfortable	14 (67%)
*Administrative Tasks*	Uncomfortable	3 (14%)
	Neutral	5 (24%)
	Comfortable	13 (62%)
3. Frequency of AI Use in Clinical/Research Activities	Never	8 (38%)
	Rarely	8 (38%)
	Montdly	1 (5%)
	Weekly	1 (5%)
	Daily	3 (14%)
4. Ways AI Tools Were Used	Generating presentation images	2 (10%)
	Automating administrative tasks	1 (5%)
	Assisting witd writing	7 (33%)
	Literature review	3 (14%)
	None	13 (62%)
5. Self-Reported Understanding of AI in Medicine	Very limited	6 (29%)
	Limited	8 (38%)
	Average	4 (19%)
	Good	1 (5%)
	Excellent	2 (10%)
6. Prior AI Education or Training	None	21 (100%)
	Grand rounds/courses/med school/specialized training	0 (0%)
7. Interest in Targeted AI Training for Nephrology	Not interested	1 (5%)
	Slightly interested	4 (19%)
	Moderately interested	3 (14%)
	Interested	4 (19%)
	Very interested	9 (43%)
8. Preferred Format for AI Training	Webinar or online lectures	3 (14%)
	Interactive workshops	11 (52%)
	Peer-led discussion groups	1 (5%)
	Self-paced online courses	3 (14%)
	Structured in-person program	3 (14%)
9. Perceived Primary Barrier to AI Adoption	Technical/infrastructure issues	2 (10%)
	Limited knowledge	12 (57%)
	Etdical/privacy concerns	0 (0%)
	Resistance to traditional practice	0 (0%)
	End-user safety concerns	7 (33%)
10. Confidence in Using AI for Clinical Decision-Making	Very uncertain	4 (19%)
	Slightly uncertain	6 (29%)
	Neutral	7 (33%)
	Confident	4 (19%)
	Very confident	0 (0%)
11. Predicted Career Impact of AI Skills	No impact	0 (0%)
	Little impact	1 (5%)
	Moderate impact	11 (52%)
	Significant impact	7 (33%)
	Crucial impact	2 (10%)
12. Overall Outlook on AI in Nephrology	Very pessimistic	0 (0%)
	Pessimistic	0 (0%)
	Neutral	3 (14%)
	Optimistic	14 (67%)
	Very optimistic	4 (19%)
13. Areas of Highest AI Potential in Nephrology	Diagnostic imaging	17 (81%)
	Predictive modeling	18 (86%)
	Patient monitoring & alerts	16 (76%)
	Personalized treatment via data analysis	12 (57%)
	Patient education & automated replies	13 (62%)
	Clinical research	10 (48%)
14. Most Interesting Starting Topic in AI	AI assistants (e.g., ChatGPT in healtdcare)	6 (29%)
	Basics of machine learning	2 (10%)
	Improving patient care witd AI tools	6 (29%)
	Using AI in medical research	1 (5%)
	Etdics and privacy	2 (10%)
	Need guidance/not sure	3 (14%)
	Otder	1 (5%)
15. AI Potential: Research *vs* Clinical Practice		
*Research – Very High*		5 (24%)
*Clinical Practice – Very High*		3 (14%)

All values are presented as number (percentage).

### Current use of AI

Despite the acknowledged relevance of AI, actual usage among the respondents was limited. Most (76%) of the respondents reported that they had never or only rarely used AI in their clinical or research activities (**Table 2**, Question 3). This disparity between the recognition of AI’s importance and its practical application highlights a gap in integration within current nephrology practice and education.

Although the majority (62%) of the respondents did not use AI-based tools, some reported the use of AI-based tools for assisting with writing tasks (33%), analyzing and reviewing medical literature (14%), generating images for presentation (10%), and automating administrative task (5%) (**Table 2**, Question 4).

### Interest in AI training

Most (67%) of the respondents reported limited or very limited understanding of AI’s capabilities in a medical context (**Table 2**, Question 5). None had formal education or training focused on AI (**Table 2**, Question 6).

The survey revealed a high level of interest in receiving targeted AI training, with 76% of the respondents expressing moderate to very high interest (**Table 2**, Question 7) (**Figure 1B**). This enthusiasm for learning more about AI technologies suggests that nephrology fellows are keen to bridge the knowledge gap and integrate AI tools into their practice.

### Preferred modes of AI training

**Figure 2:**
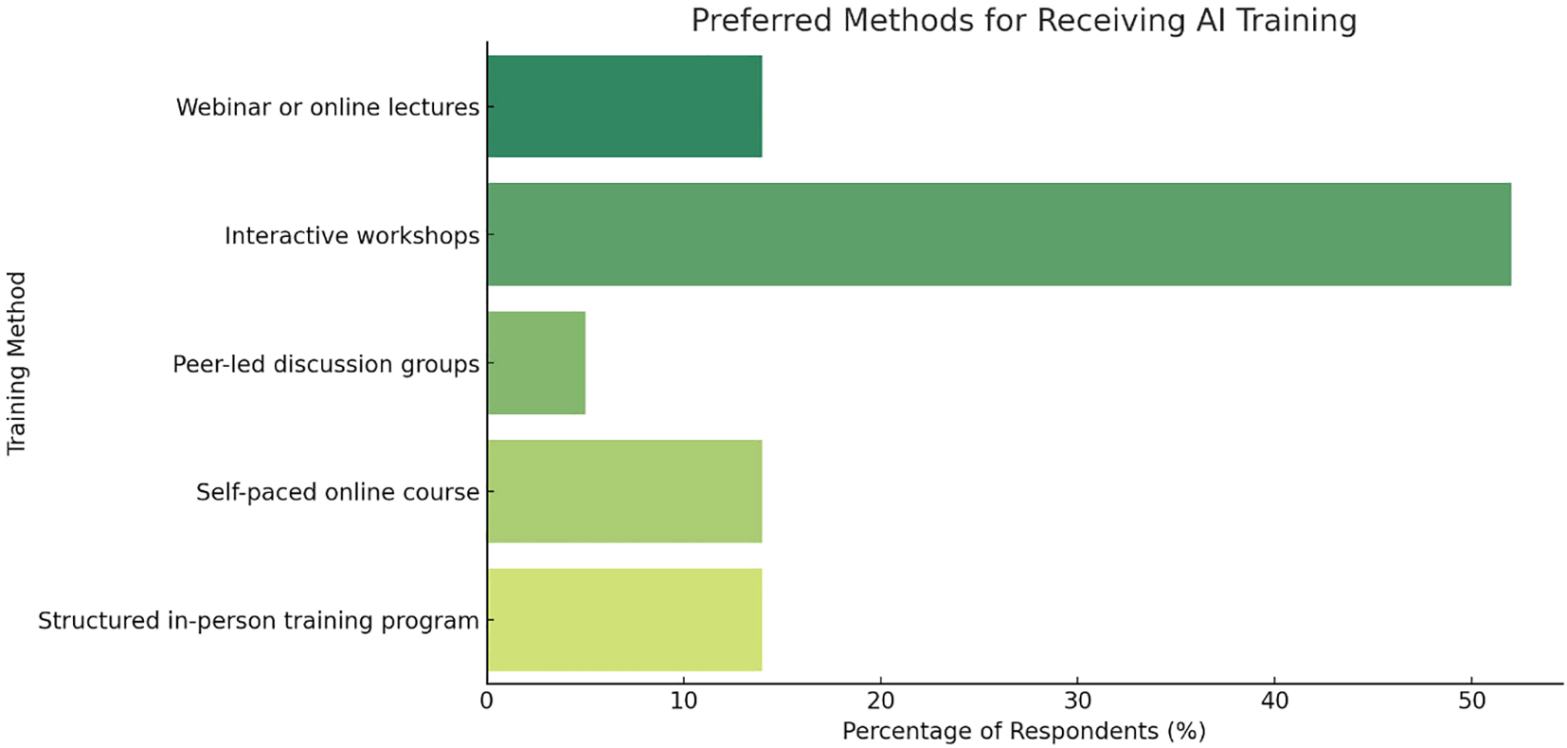
Preferred methods of AI training among nephrology fellows. Bar chart showing preferred formats for AI education. Respondents (n=21) selected their top choice among five options: webinars/online lectures, interactive workshops, peer-led discussion groups, self-paced online courses, and structured in-person training programs. Values are expressed as percentage of total responses.

Interactive workshops were the preferred format for AI training, favored by 52% of the respondents (**Table 2**, Question 8). This preference indicates a desire for hands-on, practical learning experiences that simulate real-world applications of AI in nephrology, which could help fellows gain the skills needed to utilize AI effectively in their work (**Figure 2**). In addition to interactive workshops, some respondents preferred webinars or online lectures (14%), self-paced online courses (14%), and structured in-person training programs (3%).

### Barriers to AI adoption

A significant barrier to AI adoption was limited knowledge about AI, identified by the majority (57%) of the respondents (**Table 2**, Question 9). This barrier points to a lack of sufficient AI-related content in current formal educational curricula, emphasizing the need for educational reforms to include more comprehensive AI training in nephrology fellowship programs.

### Confidence in AI for clinical decision-making

Confidence in using AI for clinical decision-making was more reserved. A substantial proportion of respondents remained neutral (33%) or uncertain (48%) about relying on AI in making clinical decisions (**Table 2**, Question 10). This cautious stance reflects concerns about the current capabilities of AI systems and the need for more robust evidence of their effectiveness and safety in clinical settings. However, almost all (95%) respondents believed AI knowledge and skill would have at least moderate to crucial impact on their future careers (**Table 2**, Question 11).

### Optimism about AI’s potential

The optimism regarding AI’s potential in nephrology was notably high among the fellows. A majority (86%) of fellows had optimistic or very optimistic overall outlooks on the integration of AI in Nephrology (**Table 2**, Question 12). Respondents thought predictive modeling (86%), diagnostic imaging and interpretation (81%), automated patient monitoring and alerts (76%), patient education and automated reply for common patient questions (62%), data analysis for personalized treatment (57%), and clinical research (48%) as areas where AI could have the most potential application (**Table 2**, Question 13).

## Discussion

Our survey study conducted among nephrology fellows at the Mayo Clinic demonstrated a robust recognition of the transformative potential of AI in nephrology, with 76% of participants considering it moderately to highly relevant. A similar proportion expressed a strong interest in AI training, even though nearly all reported minimal practical use of AI in their professional activities, highlighting a significant gap between theoretical enthusiasm and practical application. Interactive workshops were the preferred training format, and a lack of knowledge was identified as the primary barrier to AI integration. This preference underscores the demand for immersive, practice-oriented educational opportunities that bridge the gap between theory and clinical practice.

Despite high enthusiasm for AI, several key barriers hinder its adoption in nephrology fellowship training. Limited knowledge was the most frequently cited barrier (57%), followed by a lack of exposure to AI in clinical practice (76%), uncertainty in applying AI for clinical decision-making (with 33% of respondents remaining neutral and 48% uncertain), and the complete absence of formal AI training in fellowship programs (100%). These obstacles underscore a critical disconnect between the awareness of AI’s potential and the ability to harness it effectively in clinical settings. These findings highlight the urgent need for structured AI education in nephrology, including formal curricula, hands-on applications, and interdisciplinary collaborations with AI experts to enhance both confidence and competency in AI-assisted decision-making. Implementing such educational interventions is essential to transform enthusiasm into effective clinical practice.

Our findings align closely with recent developments in the field. In July, 2024, the American Society of Nephrology (ASN) launched the Partnership for Responsible AI in Kidney Health (15), an initiative aimed at creating a community of diverse stakeholders to foster a deeper understanding of AI, machine learning, and generative AI in nephrology. Notably, this initiative serves as a pivotal model for integrating responsible AI practices into clinical education, thereby reinforcing the timeliness of our survey results. This initiative, which spans clinical domains such as acute kidney injury, chronic kidney disease, dialysis, and transplant, underscores the growing recognition of AI’s importance in nephrology and the need for structured education and training, as identified in our survey.

An unexpected finding was the stark contrast between the high optimism for AI’s capabilities in predictive modeling and diagnostic imaging versus the considerable reservations regarding its use in clinical decision-making. This divergence suggests that while fellows are excited about AI’s technological potential, they remain cautious about its immediate application in clinical settings, likely due to concerns regarding its reliability and safety. These findings align with broader literature that documents both high interest in and significant hurdles to AI adoption in medical specialties (16–20). Similar studies have shown that although medical professionals are generally optimistic about the transformative potential of AI, there is widespread concern about the lack of training, data privacy issues, and the ethical implications of AI use (21, 22). However, the high rate of interest in specific, hands-on AI training modalities like workshops among nephrology fellows highlights the need for tailored educational initiatives in this specialty (23). The discrepancy between the perceived importance of AI and its limited use is primarily attributable to the current lack of formal AI education in nephrology training programs, which likely contributes to hesitancy in applying AI outputs in clinical decision-making without adequate training.

The cautious stance expressed by fellows regarding the use of AI in clinical decision-making likely reflects several layered concerns. First, many AI models, particularly those based on deep learning, function as “black boxes” with limited transparency about how decisions are generated. This lack of interpretability may reduce clinicians’ confidence in using such tools. Second, there is uncertainty surrounding accountability and medicolegal responsibility when AI tools influence patient management decisions. Third, most fellows have not been exposed to AI applications that have been rigorously validated or approved for clinical use, which likely contributes to their skepticism regarding reliability and safety. These concerns highlight the importance of not only technical education, but also structured exposure to real-world, ethically guided, and clinically validated AI applications in nephrology.

Future research should assess whether targeted educational interventions, such as workshops that simulate AI-supported clinical scenarios or training modules that explain algorithmic design and validation, can enhance fellows’ confidence over time. Longitudinal studies would be particularly valuable in measuring how educational experiences influence both perception and actual use of AI in clinical practice. Furthermore, multicenter studies involving institutions with diverse training environments and technological infrastructures could help clarify how institutional factors shape AI adoption. Comparative analyses between programs with and without integrated AI training could offer practical insights into the most effective strategies for fostering responsible and confident AI use among nephrology trainees.

The survey underscores a significant enthusiasm for AI among nephrology fellows and highlights a critical need for formal education and training programs. Bridging this gap is imperative to realize AI’s promise in enhancing patient outcomes. The current enthusiasm for AI’s potential must be matched with robust educational frameworks that not only familiarize fellows with AI technologies but also build the necessary skills to utilize these tools confidently and ethically in clinical practice. It is crucial to emphasize the potential impact of AI on patient outcomes in nephrology. AI-assisted tools, such as predictive models for CKD progression or personalized treatment recommendations, could lead to earlier interventions, improved patient care, and better overall outcomes (24, 25). This transformative potential reinforces the need to prepare fellows not only to employ AI but also to critically evaluate its applications in clinical settings. Furthermore, AI-driven tools could help address health disparities in nephrology by identifying and mitigating biases in clinical decision-making, ensuring more equitable care for all patients (26, 27). Thus, AI holds promise not only as a means to enhance efficiency but also as a vehicle to promote health equity. However, it is vital to train fellows on the potential limitations and biases of AI algorithms to prevent unintended consequences. Comprehensive training can help mitigate these risks, ensuring the ethical and effective deployment of AI technologies.

Interprofessional collaboration is essential in the development and implementation of AI tools in nephrology. Such collaborations foster an environment where clinical expertise and technological innovation synergize. Collaborations between nephrologists, data scientists, and AI experts can ensure that AI tools are clinically relevant, user-friendly, and aligned with the needs of both patients and providers (28). Encouraging fellows to engage in these collaborations during their training can foster a more integrated approach to AI adoption in nephrology. This collaborative approach not only enhances the learning experience but also accelerates the translation of AI research into clinical practice. While AI tools may automate specific tasks and improve efficiency, it is essential to emphasize that they are designed to augment, rather than replace, human expertise (29, 30). Training fellows to work alongside AI tools can help them adapt to the changing landscape of nephrology practice and ensure they remain valuable members of the healthcare team. Moreover, the lessons learned from implementing AI education in nephrology fellowship programs could serve as a model for other specialties as AI becomes increasingly integrated into various medical fields. Discussing the potential for cross-disciplinary collaboration and knowledge sharing could highlight the broader impact of this study (31).

This study’s limitations include its small sample size and single-institution focus, which may affect the generalizability of the results to all nephrology fellows or other medical specialties. Additionally, the survey method relies on self-reporting, which can introduce bias in how fellows might wish to present their familiarity with or attitudes toward AI. Future research should aim to explore the integration of AI training into nephrology curricula more broadly, potentially through multicenter studies with larger sample sizes. Longitudinal studies that can track changes in AI adoption and attitudes over time as training programs evolve would also provide valuable insights. Furthermore, exploring the impact of AI education on clinical outcomes could substantiate the need for its integration into standard medical training.

Our study achieved a strong response rate (91%) across three major campuses; however, the absolute sample size of 21 respondents presents inherent limitations. The small cohort constrains statistical power and precludes meaningful subgroup comparisons by variables such as fellowship year or subspecialty track. Moreover, the views represented may not reflect the full spectrum of experiences across nephrology training programs nationally or internationally—particularly those outside of large academic centers. Consequently, these findings should be interpreted as exploratory and hypothesis-generating. Broader generalizability will require replication in future studies with larger, more diverse samples. National and international multicenter efforts would facilitate robust subgroup analyses, allow for the detection of more nuanced patterns, and better inform the development of widely applicable AI curricula tailored to varied institutional and educational settings.

While this study includes participants from three geographically dispersed Mayo Clinic campuses—Minnesota, Arizona, and Florida—it remains institutionally anchored, and thus the findings may reflect unique institutional culture, resources, and educational priorities. Mayo Clinic has a strong research infrastructure and early AI engagement, which could influence fellows’ awareness and enthusiasm toward AI. In other settings, particularly community-based or resource-constrained fellowship programs, perceptions and access to AI-related training might differ significantly. Additionally, the demographic composition of our cohort—such as high representation of advanced subspecialty fellows (e.g., transplant and onconephrology) and likely prior academic exposure—may contribute to the relatively high interest observed. These factors underscore the importance of validating our findings through national or international multicenter studies that include a broader spectrum of training environments and demographic diversity. Such efforts would help identify contextual differences and enable tailored educational strategies across institutions.

To effectively address the educational gaps identified in this study, nephrology training programs should prioritize the development and integration of formal AI curricula that combine foundational concepts with practical application. Structured core courses should cover the basics of machine learning, natural language processing, and ethical AI use in medicine, ideally tailored to nephrology-specific applications such as AKI prediction, dialysis optimization, and transplant analytics. These courses can be delivered through modular formats (e.g., case-based lectures, flipped classrooms, or integrated bootcamps) embedded within existing academic half-days or scholarly activity blocks.

In addition to didactic components, hands-on workshops should be developed in collaboration with biomedical data scientists and informaticians. These workshops could provide fellows with real-world exposure to AI tools and platforms, such as LLMs for documentation, image interpretation software, and clinical decision support interfaces. Programs may also consider partnering with institutional data science departments or external AI education platforms to co-develop practical training modules. Furthermore, the incorporation of AI-focused quality improvement projects or electives can allow fellows to engage in applied learning within clinical or research settings.

To ensure longitudinal skill development, AI education should be scaffolded throughout fellowship training with progressive competency goals—ranging from AI literacy in the first year to applied tool evaluation and implementation strategies in the second year. Programs should also provide mentorship opportunities through faculty champions or advisory committees focused on digital health and AI integration. Ultimately, these structured and layered educational approaches are essential not only for bridging current knowledge gaps but also for preparing nephrology fellows to critically evaluate and ethically deploy AI technologies in future clinical practice.

In conclusion, the survey underscores a significant enthusiasm for AI among nephrology fellows and highlights a critical need for formal education and training programs. Structured AI education is needed to translate enthusiasm into competency, ensuring nephrology fellows can confidently apply AI in clinical settings. This study calls for educational initiatives that better prepare nephrology fellows for the impending AI integration, which is poised to significantly influence the field.

## Data Availability

The original contributions presented in the study are included in the article/**Supplementary Material**. Further inquiries can be directed to the corresponding author.
