# Supplementary material for: Artificial intelligence in nephrology education: a multicenter survey of fellowship trainees at Mayo Clinic
Source: Front Nephrol. 2025 Jun 18;5:1607017. doi: 10.3389/fneph.2025.1607017 (PMC12213394; doi:10.3389/fneph.2025.1607017)
Supplement: Supplementary file 1 [file DataSheet1.pdf]

Online Supplement

Current Perspectives and Interests in Artificial Intelligence (AI) in Nephrology 2024

Thank you for taking the time to participate in this important survey. Your input is crucial in understanding the current perspective and interest of nephrology fellows in the application and integration of Artificial Intelligence (AI) within the field. This survey seeks to gather your views on AI-related education, its relevance to nephrology, and how it can enhance clinical and research practices. This survey consists of 17 questions and will take approximately 10 minutes to complete. Your responses are completely anonymous and will be instrumental in guiding the development of AI-focused educational programs and resources tailored for nephrology.
Please complete the survey below.
Thank you!

Your expertise and opinions are highly valued, and we appreciate your contribution to this initiative.

1. **Age Group:**
   - Please select your age group:
     - 25-28
     - 29-32
     - 33-36
     - 37-40
     - >40
2. **Year in Nephrology Fellowship:**
   - Which year are you current in your nephrology fellowship?
     - First year
     - Second year
     - Third year
     - Fourth year (transplant or onco nephrology)
     - Fifth year (transplant or onco nephrology)
3. **Please select your Nephrology Fellowship Location:**
     - Mayo Clinic Arizona
     - Mayo Clinic Florida
     - Mayo Clinic Minnesota
4. **AI utilization Frequency:**
   - How often do you use AI-based tools (ChatGPT) in your clinical or research activities?
     - Daily
     - Weekly
     - Monthly
     - Rarely
     - Never
5. **AI Familiarity Level**
   - On a scale of 1-5, how would you rate your current understanding of AI’s capabilities and limitations in a medical context?
     - 1 (Very Limited): I am aware of AI but have very little understanding of how it can be applied to medicine.
     - 2 (Limited): I understand basic AI applications, such as automating simple tasks like scheduling.
     - 3 (Moderate): I understand how AI can assist with tasks like summarizing medical notes or responding to messages/emails.

    - 4 (Good): I understand how AI can be used to analyze and review large volumes of medical literature.
    - 5 (Excellent): I understand advanced AI capabilities, such as developing algorithms for predictive analytics in patient care

6. **Prior AI Education Experience**
    - Have you had any formal education or training focused on AI? If yes, please indicate the extent.
        - No formal education
        - Grand rounds
        - Comprehensive course or seminar
        - Part of undergraduate or medical school curriculum
        - Specialized training
7. **Perceived AI relevance in Nephrology**
    - Rate the current relevance of AI in nephrology on a scale of 1-5.
        - 1 (Not Relevant)
        - 2 (Slightly Relevant)
        - 3 (Moderately Relevant)
        - 4 (Relevant)
        - 5 (Highly Relevant)
8. **Interest in AI-Specific Training**
    - How interested are you in receiving targeted AI training tailored for nephrology applications, on a scale of 1-5?
        - 1 (Not Interested)
        - 2 (Slightly Interested)
        - 3 (Moderately Interested)
        - 4 (Interested)
        - 5 (Very Interested)
9. **AI integration Comfort Level**
    - On a scale of 1-5, how comfortable are you with the idea of integrating AI into your daily nephrology practice?
        - Clinical practice (summarizing previous notes, generating new notes, diagnostic assistance)
            - Uncomfortable
            - Neutral
            - Comfortable
        - Knowledge Acquisition (staying updated with the latest research, personalized learning)
            - Uncomfortable
            - Neutral
            - Comfortable
        - Administrative Tasks (scheduling, billing, patient communication)
            - Uncomfortable
            - Neutral
            - Comfortable
10. **Preferred Learning Modality for AI Training**
    - What would be your preferred method for receiving AI training?

    - Webinars or online lectures: virtual sessions focusing on theoretical aspects of AI.
    - Interactive workshops: Hands-on sessions where you can actively engage with AI tools and applications.
    - Peer-led discussion groups: collaborative learning environments for discussing AI concepts and sharing experiences.
    - Self-paced online courses: flexible learning modules that you can complete on your own pace.
    - Structured in-person training programs: comprehensive courses with a structured curriculum, covering both theory and practical applications of AI in nephrology.

11. **Perceived Barriers to AI Adoption**
    - From the following, what do you perceive as the primary barrier to the adoption of AI in nephrology?
        - Technical challenges or lack of infrastructure
        - Limited knowledge or understanding of AI.
        - Ethical or privacy concerns
        - Resistance to change in traditional practices.
        - End-user safety (accuracy of AI tools, verification of AI-generated outputs)
        - Other (please specify)
12. **Comfort with AI in Clinical Decision Making**
    - How confident are you on relying on AI for clinical decision-making in nephrology?
        - 1 (Very uncertain): I have significant doubts about the reliability and appropriateness of AI in clinical decision-making.
        - 2 (Slightly uncertain): I have some reservations about using AI for clinical decisions but am open to its potential benefits.
        - 3 (Neutral): I am undecided about the role of AI in clinical decision-making
        - 4 (Confident): I am confident in the ability of AI to assist in clinical decision-making, with appropriate human oversight.
        - 5 (Very Confident): I fully trust AI's capabilities in enhancing clinical decision-making and improving patient outcomes.
13. **Impact of AI on Career Prospects**
    - How strongly do you believe that AI knowledge and skills will impact your future career in nephrology? Rate on a scale of 1-5.
        - 1 (No Impact): I do not believe AI will significantly affect my career.
        - 2 (Little Impact): I believe AI may have a minor influence on my career, either positively or negatively.
        - 3 (Moderate Impact): I believe AI will play an important role in shaping my career, offering new opportunities.
        - 4 (Significant Impact): I believe AI will enhance my career prospects and open new avenues in nephrology.
        - 5 (Crucial Impact): AI is essential for my career advancement and will be a key factor in future success.
14. **AI Application Areas**
    - In which of the following nephrology areas do you see the most potential for AI application? Select all that apply:
        - Diagnostic imaging and interpretation
        - Patient data analysis for personalized treatment

  - Predictive modeling for disease progression
  - Automated patient monitoring and alerts
  - Clinical research and trials

15. **Preference of AI in Research or Clinical Practice**
    - If you were to start learning about AI in your field, which topic would you be most interested in exploring first? Please choose one option that interests you, and our digital health committee can provide you more education/teaching on.
      - 1 Getting to know AI assistants like ChatGPT and their use in healthcare.
      - 2 Basics of machine learning and how it helps in understanding medical data.
      - 3 Practical ways of using AI for improving patient care and treatment plans.
      - 4 Using AI tools for conducting and managing medical research.
      - 5 Ethical considerations and patient privacy in the age of AI
      - 6 I am not sure where to start, and I want guidance.
      - 7 Others, please specify in the text box below.
16. **Where do you see a greater potential for AI applications in nephrology? Rate on a scale of 1-5 for each area below:**
    - Research
      - 1 (Very Low)
      - 2 (Low)
      - 3 (Moderate)
      - 4 (High)
      - 5 (Very High)
    - Clinical Practice
      - 1 (Very Low)
      - 2 (Low)
      - 3 (Moderate)
      - 4 (High)
      - 5 (Very High)
17. **How would you describe your overall outlook on the integration of AI in nephrology?**
      - 1 (Very Pessimistic)
      - 2 (Pessimistic)
      - 3 (Neutral)
      - 4 (Optimistic)
      - 5 (Very Optimistic)
